# Genetic dissection of Arabidopsis MAP kinase phosphatase 1-dependent PAMP-induced transcriptional responses

**DOI:** 10.1093/jxb/erx335

**Published:** 2017-10-13

**Authors:** Lingyan Jiang, Ying Wan, Jeffrey C Anderson, Jie Hou, Soliman M Islam, Jianlin Cheng, Scott C Peck

**Affiliations:** 1 Department of Biochemistry, University of Missouri, Columbia, MO, USA; 2 Department of Computer Science, University of Missouri, Columbia, MO, USA; 3 Christopher S Bond Life Sciences Center, University of Missouri, Columbia, MO, USA; 4 Interdisciplinary Plant Group, University of Missouri, Columbia, MO, USA

**Keywords:** Arabidopsis, mitogen-activated protein kinase, MKP1, MPK6, pathogen-associated molecular pattern (PAMP), phosphatase, RNAseq, transcriptome

## Abstract

Plant immunity is initiated by extracellular detection of pathogen-associated molecular patterns (PAMPs) through surface-localized pattern recognition receptors (PRRs). PRR activation induces many responses including the activation of mitogen-activated protein kinases (MAPKs) that ultimately limit bacterial growth. Previous work identified Arabidopsis MAP kinase phosphatase 1 (MKP1) as a negative regulator of signaling pathways required for some, but not all, of PAMP-initiated responses. Specifically, loss of MAPK *MPK6* in an *mkp1* background suppressed a subset of the *mkp1*-dependent biological phenotypes, indicating the requirement for MPK6 in MKP1-dependent signaling. To further genetically separate the outputs of PAMP-responsive signaling pathways, we performed a transcriptome analysis in Arabidopsis wild type, *mkp1* and *mkp1 mpk6* seedlings treated with the bacterially derived PAMP elf26 for 0, 30, and 90 min. Using differential genetic and temporal clustering analyses between and within genotypes, we identified and separated 6963 elf26-responsive transcripts based on both genetic requirements of MKP1 (with or without a requirement for MPK6) and temporal transcriptional accumulation patterns, and some of these novel response markers were validated by qRT-PCR over a more extended time course. Taken together, our transcriptome analysis provides novel information for delineating PAMP signaling pathways.

## Teaser

Clustering of PAMP-induced transcripts regulated by Arabidopsis MAP kinase phosphatase 1 delineates signaling pathways that are MKP1 dependent and MPK6 dependent and independent, providing a validated set of molecular pathway markers.

## Introduction

To protect themselves against potential bacterial pathogens, plants need to activate immune responses rapidly and effectively when invading microbes are detected. Bacteria initially are perceived through the recognition of pathogen-associated molecular patterns (PAMPs) such as the flagellin protein required for motility (Felix *et al.*, 1999), lipopolysaccharides from the bacterial cell wall (Desaki *et al.*, 2006), and the bacterial translation elongation factor EF-Tu (Zipfel *et al.*, 2006). This non-self recognition is mediated by specific plasma membrane-localized pattern recognition receptors (PRRs) that initiate a diverse array of signaling events including reactive oxygen species (ROS) production, cellular Ca^2+^ influx, reversible protein phosphorylation, and transcriptional reprogramming (Boller and Felix, 2009). Some or all of these immune responses ultimately restrict the growth of the bacterial invader, contributing to the basal defense program called pattern-triggered immunity (PTI).

Transcriptional profiling of plant tissue treated with different single elicitors has identified sets of transcripts that change in response to diverse elicitors in Arabidopsis cell culture or seedlings (Navarro *et al.*, 2004; Zipfel *et al.*, 2004, 2006; Ramonell *et al.*, 2005; Bae *et al.*, 2006; Qutob *et al.*, 2006; Denoux *et al.*, 2008). These studies demonstrated considerable overlap in response to multiple elicitors, indicating that different elicitors activate conserved basal defense responses (Jones and Dangl, 2006). In addition, they also found that the most abundant categories of gene products encoded by PAMP-responsive genes included signaling components such as transcription factors, protein kinases and/or phosphatases, and proteins that regulate protein turnover (Navarro *et al.*, 2004; Zipfel *et al.*, 2004, 2006; Denoux *et al.*, 2008). Recently, a high-resolution time course study investigated genome-wide expression changes following challenge with the virulent pathogen *Pseudomonas syringae* pv tomato DC3000 (DC3000) and the non-pathogenic mutant strain DC3000 *hrpA−*. This work captured gene expression dynamics of PTI induced by DC3000 *hrpA−* and how these expression profiles were modulated by DC3000 (Lewis *et al.*, 2015). However, time course studies that only involve treatment of wild type plants do not provide any immediate information on the specific pathways connecting perception from the plasma membrane localized receptor(s) to specific downstream transcriptional changes.

Mitogen-activated protein kinases (MAPKs) and calcium-dependent protein kinases (CDPKs) are signaling components that link the perception from PRR complexes to downstream defense responses (Tena *et al.*, 2011; Li *et al.*, 2016). Studies of innate immunity in Arabidopsis mainly focus on three MAPKs: MPK3, MPK6, and MPK4. MPK3 and MPK6 appear to be partially redundant and are activated by the upstream MAPK kinases MKK4/MKK5, whereas MPK4 is regulated by MKK1/MKK2 (Nühse *et al.*, 2000; Asai *et al.*, 2002; Suarez-Rodriguez *et al.*, 2007; Qiu *et al.*, 2008). A comparative transcriptome analysis of *mpk3*, *mpk4*, and *mpk6* mutants revealed that 36% of the flg22-up-regulated genes and 68% of the flg22-down-regulated genes were affected in at least one of the *mpk* mutants, pointing out the essential role of these MAPKs in regulating the transcriptional reprogramming during PAMP signaling (Frei dit Frey *et al.*, 2014). A separate study using constitutively active forms of two CDPKs, CPK5 and CPK11, transiently expressed in the protoplasts identified target genes downstream of these kinases (Boudsocq *et al.*, 2010). Adding a layer of complexity, MAPKs and CDPKs may act either alone or synergistically in controlling PAMP-induced transcript accumulation. By transiently expressing constitutively active CPK5 and/or MKK4 in protoplasts, several marker genes for CDPK-specific, MAPK-specific and CDPK–MAPK synergistic pathways were identified (Boudsocq *et al.*, 2010), and these few markers have been widely used to begin delineating the PAMP signaling pathways by placing newly discovered putative signaling components into different pathways (Desclos-Theveniau *et al.*, 2012; Lozano-Durán *et al.*, 2013; Prince *et al.*, 2014; Smith *et al.*, 2014; Domínguez-Ferreras *et al.*, 2015). The utility of just this small number of pathway markers clearly demonstrates how identifying additional transcripts regulated by separate pathways can assist the field in better molecular characterization of genetic mutants with altered defense responses. Therefore, the goal of this study was to make use of a novel set of genetic mutants to define a new set of molecular pathways.

Protein phosphatases are important components required for the proper regulation of PAMP signaling. Phosphatases often act as negative regulators that function by dephosphorylating and inactivating kinases, including MAPKs, to prevent overactive stimulation of defense responses. In a screen for phosphatases involved in the proper regulation of PAMP responses, we uncovered MAPK phosphatase 1 (MKP1) as an important negative regulator as evidenced by heightened early responses to PAMP elicitation in the *mkp1* null mutant such as enhanced MAPK activation, ROS production, and transcript accumulation of some but not all PAMP-regulated genes (Anderson *et al.*, 2011). In addition, later PAMP responses such as seedling growth inhibition and resistance against normally virulent DC3000 were also enhanced in the *mkp1* mutant. Interestingly, these responses were suppressed in *mkp1 mpk6* double mutants but not in *mkp1 mpk3* mutants, indicating that enhanced biological responses (i.e. resistance and growth inhibition) in *mkp1* specifically require MPK6 but not MPK3 (Anderson *et al.*, 2011). The results were consistent with the fact that although MKP1 could interact at least somewhat with MPK3 and MPK4, it showed a much stronger preference for MPK6 (Ulm *et al.*, 2002). More recently, we found that the enhanced resistance against DC3000 in *mkp1* can be explained by the decreased abundance of specific extracellular metabolites from the plant that DC3000 uses as signals to activate its virulence program (Anderson *et al.*, 2014). Interestingly, during this study we found that while all the bioactive metabolites were restored to wild type (WT) levels in *mkp1 mpk6* plants, some of the non-bioactive metabolite levels altered in *mkp1* were not affected by the loss of MPK6 (Anderson *et al.*, 2014). These results provided the first evidence that not all *mkp1*-dependent changes involve MPK6, indicating the existence of MKP1-dependent, MPK6-independent pathways. Thus, it seemed likely that these mutants that affect early signaling responses would be useful tools to genetically separate different signaling pathways leading to specific changes in transcript accumulation.

In this study, we compared transcript accumulation profiles of Arabidopsis seedlings of wild type (Ws), *mkp1* (Ws), and *mkp1 mpk6* (Ws) treated with elf26 (a 26-amino acid peptide corresponding to a conserved domain of bacterial elongation factor EF-Tu) at 0, 30, and 90 min post-treatment. By differential expression analysis, we separated the PAMP-responsive transcripts into defined genetic pathways. In addition, by clustering transcripts within discrete temporal accumulation profiles, we also predicted genes that are likely targets of different transcriptional regulation within these genetic pathways. Gene Ontology (GO) analysis indicated distinct biological processes that are specifically associated with MKP1-dependent genes. Finally, the expression patterns of selected marker genes were confirmed by quantitative real-time PCR over a more extended time course following elicitation, providing a validated set of novel molecular pathway markers for plant defense studies.

## Materials and methods

### Plant material

Arabidopsis ecotype Wassilewskija (Ws) was used in this study. The mutants *mkp1-1* (Ws) and *mkp1-1 mpk6-1* (Ws) have been described previously (Ulm *et al.*, 2001; Liu and Zhang, 2004; Bartels *et al.*, 2009; Anderson *et al.*, 2011, 2014) and also have been confirmed by genotyping (Supplementary Fig. S1 at *JXB* online). Seeds were sterilized with 1% sodium hypochlorite and 0.01% Tween-20 for 20 min, rinsed with water, and plated aseptically on 0.5% agar containing 2.1 g l^−1^ Murashige and Skoog (MS) salts (PhytoTechnology Laboratories, http://www.phytotechlab.com/), pH 5.7, 1% sucrose and 6.4 g ml^−1^ MS salts vitamin powder (PhytoTechnology Laboratories). After stratification for 2 d at 4 °C, seeds were grown at 22 °C with a 16/8 h light–dark cycle and 70% humidity. Seedlings were maintained in the same growth chamber under the same conditions during elf26 treatments.

### Transcriptome studies

Prior to elicitation, 12-day-old seedlings of Ws, *mkp1*, *mkp1 mpk6* were transferred from MS agar plates to incubate in sterile water overnight for 16–20 h. For elicitor treatments, water from the overnight incubation was removed and replaced with sterile water containing 1 µM elf26. Seedlings were harvested at 0, 30, and 90 min post-treatment. Three independent experiments that included each genotype and time point were performed. Total RNA was isolated with the RNeasy Plant Mini Kit (Qiagen, http://www.qiagen.com/). RNAseq libraries were prepared using the TruSeq RNA sample preparation kit (Illumina) following the manufacturer’s protocol (Illumina, http://www.illumina.com). All samples were sequenced by an Illumina HiSeq 2500 at DNA core in Bond Life Sciences Center, University of Missouri–Columbia. Raw reads were filtered using the NGS QC tool kit (Patel and Jain, 2012). The first 13 bp from the 3′ end of the reads, which showed unstable base composition as determined by the percentage of four different nucleotides (A, T, C, and G) and low quality bases [PHRED-like score (*Q* score)<20], were trimmed. The reads containing primer/adaptor sequences and low quality reads (the percentage of low quality bases ≥30%) were removed. All high-quality reads from all biological experiments were mapped to Arabidopsis TAIR 10 genome with TopHat (http://tophat.cbcb.umd.edu/), and only the uniquely mapped reads were chosen for differential gene analysis. Transcript abundance and differential gene expression were calculated with the Cufflinks package (Trapnell *et al.*, 2012). Specifically, the mapped reads were assembled into transcripts by Cufflinks (http://cufflinks.cbcb.umd.edu/). The resulting assemblies were merged using Cuffmerge. Aligned reads and merged assemblies were used to calculate expression levels and to determine statistical significance using Cuffdiff. Transcript abundance of each gene was estimated by fragments per kilo base of transcript per million fragments mapped (FPKM). The raw Illumina reads generated from RNAseq experiments were deposited at NCBI Sequence Read Archive (SRP101277).

### Differential gene expression analysis

Transcripts with significant changes (absolute value of log_2-fold_ change ≥1, *q*≤0.01) in Ws or *mkp1* post-30 min and/or 90 min elf26 elicitation compared with 0 min were considered to be elf26-responsive transcripts. Transcripts with at least 1.5-fold difference (*q*≤0.01) between Ws and *mkp1* in the same treatment condition were considered to be MKP1 dependent. The MKP1-independent transcripts were defined as the set of total elf26-responsive transcripts with MKP1-dependent transcripts subtracted. Within the MKP1-dependent category, transcripts with a significant (*q*≤0.01) difference between *mkp1* and *mkp1 mpk6* but no significant difference (*q*>0.01) between Ws and *mkp1 mpk6* post-elf26 elicitation were considered to be both MKP1 and MPK6 dependent. Transcripts with no significant difference (*q*>0.01) between *mkp1* and *mkp1 mpk6* but significant difference (*q*≤0.01) between Ws and *mkp1 mpk6* were MKP1 dependent/MPK6 independent. MKP1-dependent transcripts that do not meet either criterion were considered to be partially MPK6 dependent.

### Clustering analysis

The dataset for the co-expression analysis was built from the results of the differential transcript abundance analyses. Transcripts that differentially accumulated at either 30 min or 90 min post-elf26 elicitation were included for the analysis; and the clustering was performed with STEM (Short Time-series Expression Miner) using the temporal profiles in Ws (Ernst and Bar-Joseph, 2006). Default values were selected for two variable parameters: 50 as maximum number of model profiles and 2 as maximum unit change in model profiles between time points and combined clusters with similar general trends. Cluster profiles were graphed with the R package and represented as line graphs in three groups based on the different scale ranges (low, medium, high).

### Validation using quantitative real-time PCR

Elicitation treatment was performed using the same conditions as described for the transcriptome analysis. For each time point, seedlings were blotted dry and frozen in liquid nitrogen. Total RNA was isolated using TRI reagent (Sigma-Aldrich) and treated with DNase I (Thermo Fisher Scientific) before 1 µg of RNA was reverse-transcribed in 25 µl reactions containing 5 µM DTT, 0.5 µl RnaseOUT (Thermo Fisher Scientific), 2 µM oligo(dT), 1 mM each of dNTPs and 0.5 µl M-MLV reverse transcriptase (Promega, http://www.promega.com/) for 1 h at 42 °C, followed by 5 min at 85 °C. Reverse transcription reactions were diluted to 100 µl using diethylpyrocabonate-treated water. Real-time PCR reactions were performed using primers listed in Supplementary Table S1. Briefly, 10 µl real-time PCR reactions containing 5 µl SYBR Green PCR master mix (Thermo Fisher Scientific), 1 µl cDNA and 0.2 µM of each primer were performed using an ABI7500 real-time thermal cycler (Thermo Fisher Scientific). Three independent experiments were performed. Expression levels were calculated using the equation: expression level=(PCR efficiency)^−Δ*C*t^, where Δ*C*_t_=*C*_t_(sample)−*C*_t_(control), and the cycle threshold *C*_t_ and PCR efficiency for each reaction were obtained using the software program LINREGPCR (Ramakers *et al.*, 2003). *At2g28390* (SAND family protein) was used as the reference gene for normalizing *C*_t_ values (Czechowski *et al.*, 2005). Statistical analyses were performed using Student’s two-sample unpaired *t* tests.

### Gene Ontology analysis

The web-based agriGO software (http://bioinfo.cau.edu.cn/agriGO/index.php) was used to assign GO functional categories. Singular enrichment analysis was used to compute over-represented categories in the sets of MKP1-dependent genes by comparing them with GO terms in the set of elf26-up-regulated genes or elf26-down-regulated genes using Fisher’s exact test (Du *et al.*, 2010). Over-represented GO terms in the MKP1-dependent genes, elf26-responsive genes as compared with all expressed genes in the whole Arabidopsis genome were also calculated. The cut-off (*q*≤0.05) was used for significantly over-represented GO terms.

## Results and discussion

### Identification of transcriptomes of Ws, *mkp1*, and *mkp1 mpk6* in response to elf26

MKP1 is a negative regulator of multiple PAMP-induced defense responses in Arabidopsis (Anderson *et al.*, 2011). In a limited characterization of a few early defense related genes, only six out of eight PAMP-induced transcript profiles were altered in *mkp1*, indicating that MKP1 regulates some, but not all, transcriptional pathways (Anderson *et al.*, 2011). Loss of function mutations in *MPK6* but not in *MPK3* suppress a number of the molecular and biological phenotypes of the *mkp1* mutant, including the enhanced resistance against DC3000 and PAMP-induced growth inhibition, indicating that MPK6 but not MPK3 acts within MKP1-dependent signaling pathways leading to enhanced resistance (Anderson *et al.*, 2011). A subsequent study showed that all biologically active metabolites involved in the enhanced resistance in the *mkp1* mutant are restored to wild type levels in the *mkp1 mpk6* double mutant (Anderson *et al.*, 2014). However, some of the non-bioactive extracellular metabolites with altered abundance in *mkp1* were not restored in the *mkp1 mpk6* double mutant (Anderson *et al.*, 2014). These observations indicated two important findings: (i) not all MKP1-regulated phenotypes are MPK6 dependent; and (ii) The use of the *mkp1 mpk6* double mutant can be used to eliminate molecular responses that are not directly correlated with resistance in *mkp1*. Therefore, it was likely that not all *mkp1*-dependent changes in PAMP-regulated transcript accumulation patterns would require MPK6; and the identification of *mkp1*-dependent changes that also required MPK6 would define transcript changes most associated with the enhanced resistance.

To genetically separate PAMP-responsive transcripts into MKP1-dependent and MKP1-independent categories, as well as to further subdivide the MKP1-dependent transcripts into MPK6-dependent and MPK6-independent pathways, we used RNAseq to perform a whole transcriptome analysis of Ws, *mkp1*, and *mkp1 mpk6* seedlings treated for 0, 30, or 90 min with 1 μM elf26. For transcriptome sequencing, a total of 737 407 322 reads from all biological experiments passed the quality filter and were mapped to the Arabidopsis TAIR10 genome. Approximately 89.29% of high-quality reads mapped uniquely to a single annotated TAIR10 gene (Supplementary Table S2). These high-quality data were then analysed by different comparisons as described below.

### Identification of MKP1-dependent transcripts in elf26-triggered transcriptional reprogramming

First, transcriptomes of Ws and the *mkp1* mutant in response to 30 and 90 min treatment with elf26 were compared; and transcripts that showed significant (*q*≤0.01) changes in response to elf26 in Ws and *mkp1* mutant were considered to be elf26-responsive. At 30 min, 2895 genes had altered transcript levels (≥2-fold) after elf26 treatment. By 90 min, the response to elf26 increased to include 5989 transcripts with altered levels. Among all elf26-responsive transcripts at either time point, 5109 were induced by elf26, whereas 3775 were repressed (Supplementary Table S3; for a complete gene list including normalized expression values, log2-fold change (Fc), *q*-values and annotations, see Supplementary Table S4).

**Fig. 1.**
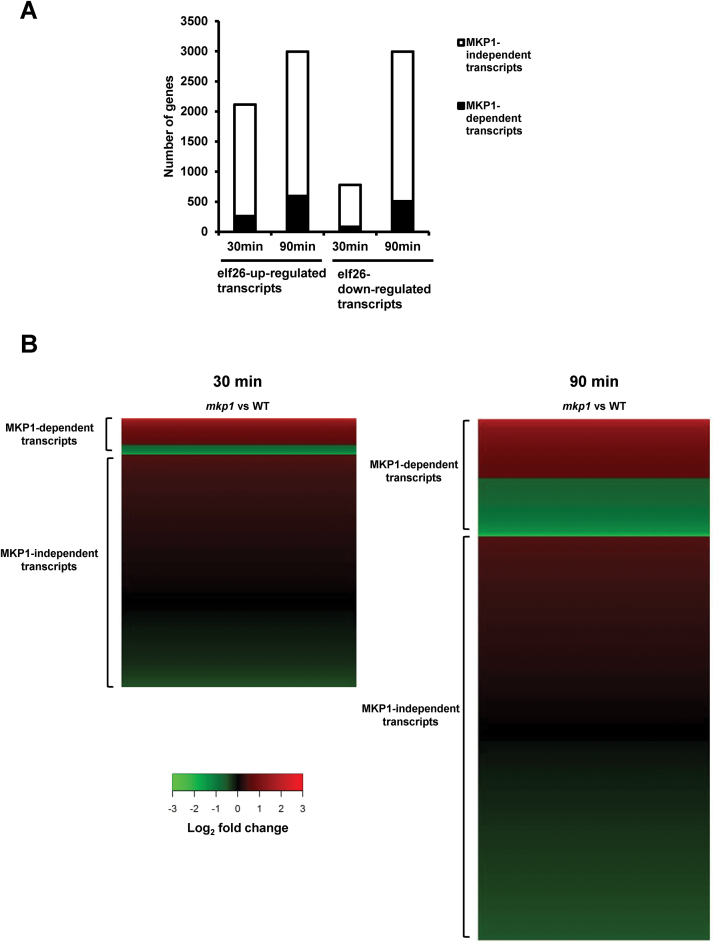
Only a subset of PAMP-regulated transcripts is MKP1 dependent at 30 and 90 min post-elicitation. (A) The total PAMP-regulated (elf26) transcripts are divided into transcripts differentially accumulating in *mkp1* mutant (black bars) and transcripts not affected by *MKP1* mutation (white bars). (B) These are also are shown represented by a heat map.

Transcripts with at least 1.5-fold difference between the *mkp1* mutant and Ws were considered to be differentially accumulating in *mkp1* and, therefore, MKP1 dependent. At 30 min post-elicitation, we observed that 12% (262/2114) of the elf26-induced transcripts and 11% (85/785) of elf26-repressed transcripts were affected in the *mkp1* mutant (Fig. 1; for a complete gene list see Supplementary Table S5). At 90 min, the proportion of MKP1-dependent transcripts increased to 20% (594/2995) of elf26 up-regulated and 17% (508/2994) of elf26-down-regulated transcripts. Altogether, these results were consistent with the previous limited study (Anderson *et al.*, 2011) indicating that the abundance of only a subset of PAMP-responsive transcripts is regulated by MKP1. We should note that the comparison of untreated samples indicated that some of the MKP1-dependent differences exist between transcripts at 0 min, and these are summarized in Supplementary Table S5. However, these small changes did not significantly contribute to the quantitative differences between genotypes following elf26 treatment; so all elf26-induced differences result from the stimulus rather than any pre-existing differences within the genotypes.

### Gene Ontology characterization of MKP1- dependent genes

**Fig. 2.**
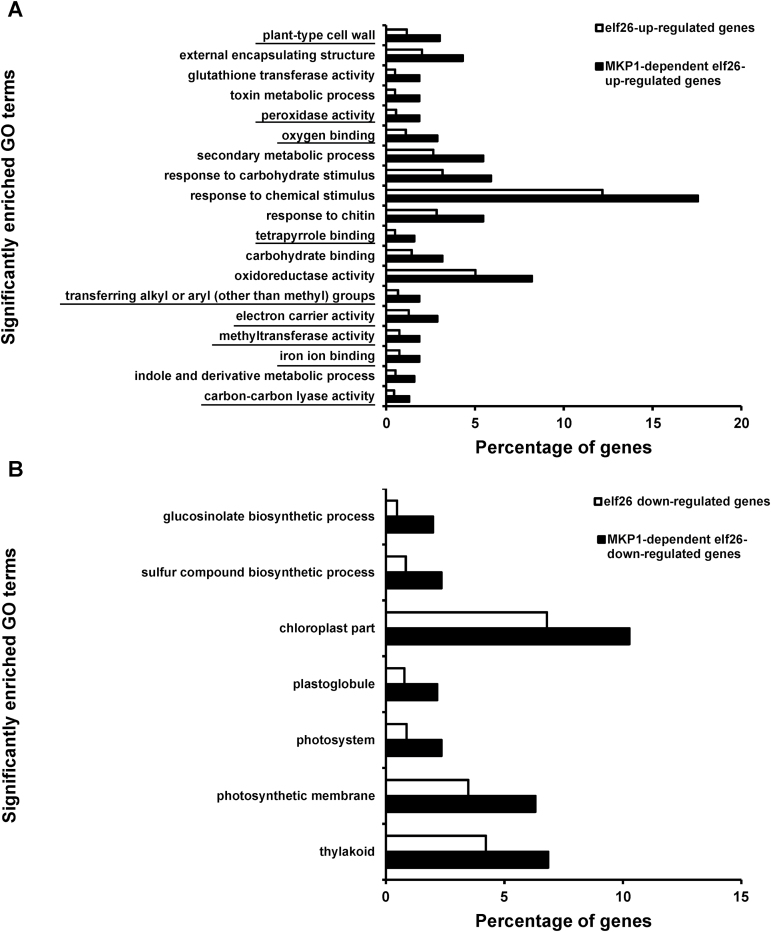
MKP1-dependent genes show significant enrichment in discrete GO categories. Significantly enriched GO terms (*q*≤0.05) of MKP1-regulated genes (black bars) compared with all elf26-regulated genes (white bars) for both up-regulated genes (A) and down-regulated genes (B). The underlined GO terms are uniquely enriched in the MKP1-dependent genes (not in elf26-regulated genes) compared with the whole Arabidopsis genome.

To investigate the biological processes that are over-represented in MKP1-dependent genes, Gene Ontology (GO) analysis with agriGO was performed (Du *et al.*, 2010). First, over-represented GO categories within MKP1-dependent genes in comparison with all elf26-regulated genes were found by singular enrichment analysis. We observed that 19 GO categories were significantly (*q*≤0.05) over-represented in the MKP1-dependent elf26 up-regulated genes, whereas seven GO terms were enriched in the MKP1-dependent elf26 down-regulated genes (Fig. 2, Supplementary Table S6). In addition, GO terms significantly (*q*≤0.05) enriched in MKP1-dependent genes and in all elf26-regulated genes were also compared with the whole Arabidopsis genome (Supplementary Table S6). Most of the GO enriched terms in the *mkp1* mutant were similarly enriched in the set of elf26-responsive genes. However, by comparing the GO terms enriched in elf26-regulated genes with those in MKP1-dependent genes, we observed that nine GO terms were uniquely enriched in *mkp1* as compared with the whole genome (underlined in Fig. 2). Within the categories uniquely enriched in *mkp1*, we found GO terms related to multiple defense responses such as cell wall modification, redox reactions, and iron binding. Previous studies found that rapidly PAMP-induced genes were functionally enriched for enzymes involved in antimicrobial compound biosynthesis and for proteins involved in signal perception and transduction (Navarro *et al.*, 2004; Zipfel *et al.*, 2004, 2006; Denoux *et al.*, 2008; Frei dit Frey *et al.*, 2014). By contrast, genes associated with photosynthesis-related process, fatty acid metabolism and glucosinolate biosynthesis were significantly repressed by PAMPs, suggesting that plants may redirect secondary metabolism and reduce the production of photosynthates to restrict the resources required for pathogen growth (Lewis *et al.*, 2015). By comparison, our GO analysis of MKP1-dependent genes indicates that some biological processes enriched in MKP1-dependent genes are unique (i.e. not enriched in wild type elf26-responsive genes), and that some MKP1-dependent overlap with ones normally occur during PTI signaling, with the representation of these processes being further amplified. These distinct patterns of transcript accumulation suggest that loss of MKP1 may lead to aberrant signaling outputs that are not typically observed in wild type seedlings, as well as the hyper-response of a small subset of genes that are normally PAMP responsive.

### Subdivision of MKP1-dependent transcripts into MPK6-dependent and MPK6-independent categories

**Fig. 3.**
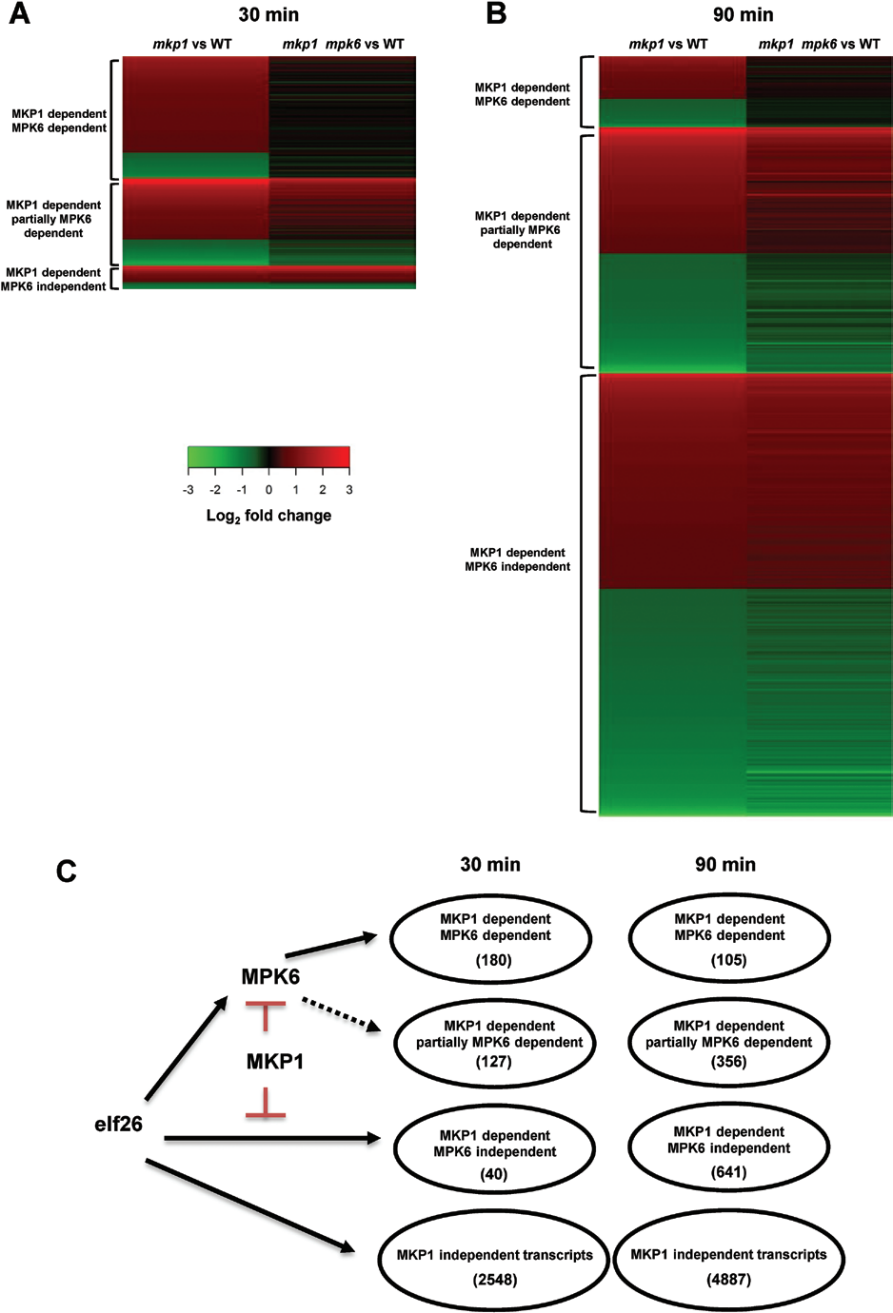
MKP1 plays a complex role in the regulation of PAMP-responsive transcripts. (A, B) Heat maps show the log2-fold change in transcript accumulation level between indicated comparison of genotypes. (C) Numbers of transcripts in different genetic categories.

We next investigated if MKP1-dependent transcripts could be further subdivided into MPK6-dependent or MPK6-independent pathways using comparisons with responses in the *mkp1 mpk6* double mutant. MKP1-dependent transcripts were considered MPK6 dependent if the changes in the *mkp1* mutant were completely reversed to being indistinguishable from WT levels in the *mkp1 mpk6* double mutant. Conversely, transcripts were considered MPK6 independent if the transcript levels in the *mkp1 mpk6* double mutant were not significantly (*q*>0.01) different from those in the *mkp1* mutant but were significantly (*q*≤0.01) different from those in Ws. Heat map analyses show the log_2_-fold change ratio in gene expression between different genotypes (Fig. 3A, B). Transcripts not discreetly meeting either of these criteria were considered to be partially MPK6 dependent and not included in the heat map (Supplementary Table S7 compiles all data including the transcripts in different genetic categories at different time points post-elicitation).

Using these criteria, we found that at 30 min post-elf26 treatment, 180 (82%) of the MKP1-dependent transcripts were also dependent on MPK6, whereas only 40 (18%) of the MKP1-dependent transcripts were independent of MPK6. However, at 90 min the pattern flipped, with 86% (641) of the MKP1-dependent transcripts being MPK6 independent with only 14% (105) being MPK6 dependent (Fig. 3C). These observations indicate that MPK6 plays a more significant role in MKP1-dependent transcript accumulation during early PAMP responses.

Previous quantitative RT-PCR results showed that six out of eight PAMP-responsive transcripts accumulated to higher levels but none to lower levels (the other two were unchanged) in the *mkp1* mutant compared with WT, indicating that MKP1 acts primarily as a negative regulator in controlling elf26-responsive transcript accumulation (Anderson *et al.*, 2011). The results of the current comprehensive study are consistent with these initial observations, with 80% (143/180) of the transcripts in the MKP1-dependent/MPK6-dependent category showing hyper-induction in the *mkp1* mutant compared with WT at 30 min elf26 treatment (Fig. 3A, Supplementary Table S7). However, this study also identified a subset of PAMP-responsive transcripts with lower accumulation in *mkp1* (Fig. 3). Moreover, the proportion of transcripts with either increased or decreased level in the *mkp1* mutant was close to equivalent at 90 min, indicating a more complex role of MKP1 in regulating PAMP-responsive transcription as responses progress (Fig. 3B, Supplementary Table S7).

Because there are only five MAPK phosphatases in Arabidopsis but 20 MAPKs (Colcombet and Hirt, 2008), it is likely that MKP1 regulates pathways involving other MAPKs. MKP1 was previously shown to interact with both MPK3 and MPK4 (Ulm *et al.*, 2002). In addition, MPK3 was also hyperactivated in *mkp1* mutants along with MPK6 during elf26 treatment (Anderson *et al.*, 2011). Therefore, it is quite likely that at least a portion of the MKP1-dependent but MPK6-independent transcript changes are regulated by MPK3. However, we cannot rule out that additional MAPKs such as MPK1, MPK11, or MPK13 (Nitta *et al.*, 2014) may also contribute to these MKP1-dependent changes in transcript accumulation.

### Temporal clustering analysis to predict putative co-regulated genes

The differential analysis performed on the transcriptome data between genotypes separated transcripts based on statistically significant differential accumulation at specific time points but did not consider the accumulation pattern across different time points. Genes with similar accumulation profiles may share a regulatory protein (e.g. transcription factor) or mechanism, possibly placing the transcripts in the same signaling pathways. To identify transcripts that behave similarly during elicitation, we performed a co-expression analysis using the STEM (Short Time-series Expression Miner) tool because of its effectiveness in clustering short time-series data (Ernst and Bar-Joseph, 2006). We performed this analysis with PAMP-responsive transcripts and separated them based on their temporal profiles in the wild type, because only the magnitude of the PAMP-responsive transcripts and not the temporal accumulation pattern was altered in the *mkp1* mutant (i.e. the patterns were the same as in wild type).

**Fig. 4.**
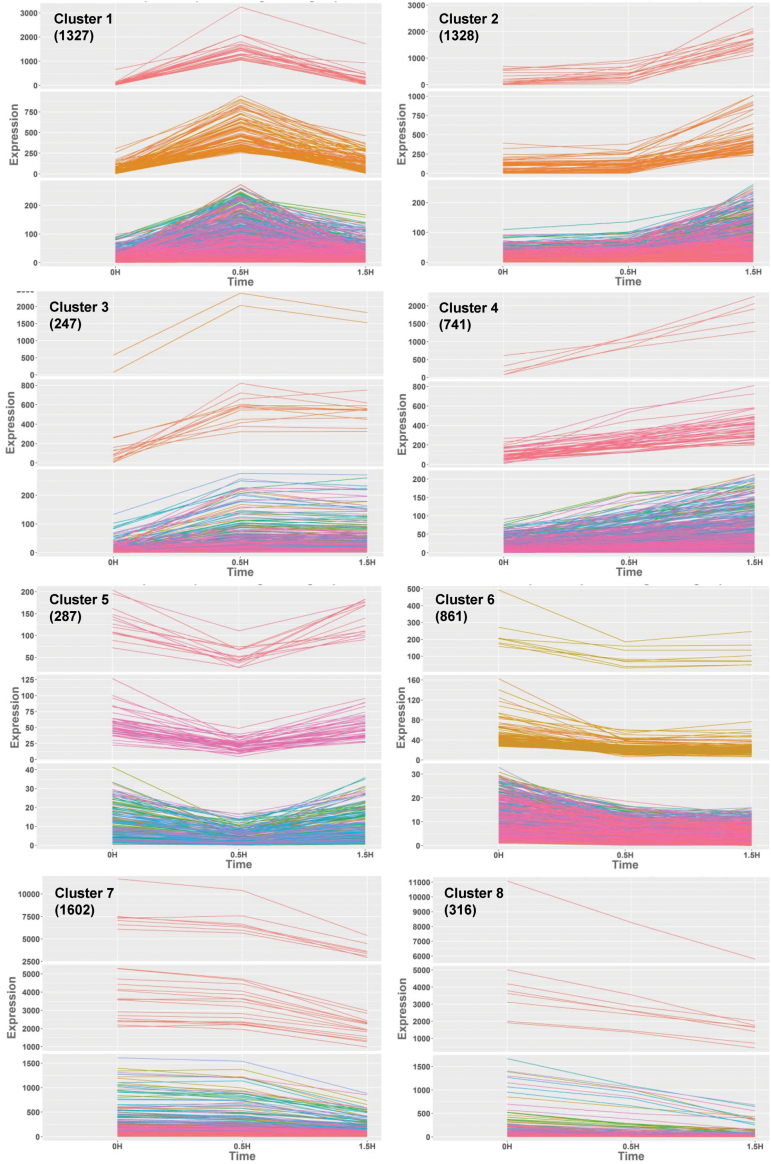
PAMP-responsive genes can be clustered based on temporal expression patterns. PAMP-responsive genes were clustered by STEM (Short Time-series Expression Miner) according to their expression kinetics in wild type plants. Eight major clusters were obtained. The temporal expression profiles of genes in each cluster are represented by line graphs (the top, middle, and bottom graphs of each cluster represent transcripts separated by high, medium, and low expression levels, respectively). The *y*-axis of each graph is the normalized expression value of each gene in artificial units, and the *x*-axis is the corresponding time points after elf26 treatment. The numbers in parentheses are the number of genes within each cluster. (This figure is available in color at *JXB* online.)

According to the clustering algorithm from STEM, 96% (6709/6964) of the PAMP-responsive transcripts could be assigned to a single model profile (the remaining could not be confidently assigned to a single category). This analysis yielded eight major group clusters (the three different graphs are different scales to allow visualization of all genes with similar patterns): (i) early induced and transient (cluster 1); (ii) late induced (cluster 2); (iii) early induced and sustained (cluster 3); (iv) early induced and amplified (cluster 4); (v) early repressed and transient (cluster 5); (vi) early repressed and sustained (cluster 6); (vii) late repressed (cluster 7); (viii) early repressed and amplified (cluster 8) (Fig. 4; for a complete list of genes associated with each cluster, see Supplementary Table S8). To investigate the possible correlation between accumulation kinetics and genetically distinct pathways, we determined the representation of different clusters within the genotype-specific response categories. In general, we did not observe a unique correlation between a type of transcript accumulation pattern with a specific genetic category, indicating that the regulation of MKP1 and or MPK6 is not restricted to transcripts within a certain type of accumulation pattern but widely covers transcripts displaying different types of temporal profiles. However, we observed that MKP1-dependent transcripts showed a higher representation in several clusters. For instance, in cluster 7 (late repressed) at 30 min post-elf26 treatment, the percentage of MKP1-dependent transcripts was 75 times that of MKP1-independent transcripts. At 90 min in clusters 5 (early repressed and transient) and 8 (early repressed and amplified), the proportion of MKP1-dependent transcripts was almost five times that of MKP1-independent transcripts. Because clusters 5, 7 and 8 were all associated with elf26-repressed transcripts, these results indicate an important role of MKP1 in regulating the abundance of elf26-repressed transcripts as well as elf-induced transcripts (Table 1).

**Table 1.** Percentage of cluster in each genetic category

Cluster	Description	30 min			90 min		
		MKP1- independent transcripts	MKP1-dependent MPK6-dependent transcripts	MKP1-dependent MPK6-independent transcripts	MKP1- independent transcripts	MKP1-dependent MPK6-dependent transcripts	MKP1-dependent MPK6-independent transcripts
1	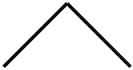	44.58% (1136/2548)	46.67% (84/180)	30.00% (12/40)	9.94% (486/4887)	12.38% (13/105)	20.44% (131/641)
2	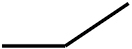	6.75% (172/2548)	10.00% (18/180)	10.00% (4/40)	22.88% (1118/4887)	21.90% (23/105)	14.51% (93/641)
3	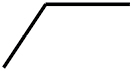	7.73% (197/2548)	12.22% (22/180)	17.50% (7/40)	3.56% (174/4887)	3.81% (4/105)	5.77% (37/641)
4	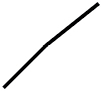	13.23% (337/2548)	8.33% (15/180)	25.00% (10/40)	12.11% (592/4887)	13.33% (14/105)	12.48% (80/641)
5	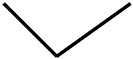	9.50% (242/2548)	9.44% (17/180)	2.50% (1/40)	0.90% (44/4887)	2.86% (3/105)	1.87% (12/641)
6	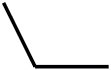	12.72% (324/2548)	8.89% (16/180)	5.00% (2/40)	14.22% (695/4887)	12.38% (13/105)	11.23% (72/641)
7	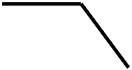	0.04% (1/2548)	0.56% (1/180)	2.50% (1/40)	28.14% (1375/4887)	20.95% (22/105)	20.28% (130/641)
8	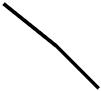	2.63% (67/2548)	2.22% (4/180)	2.50% (1/40)	4.01% (196/4887)	11.43% (12/105)	12.32% (79/641)
**Total**		97% (2476/2548)	98% (177/180)	95% (38/40)	95% (4680/4887)	99% (104/105)	99% (634/641)

### Validation of unique transcripts that are representative of different regulatory pathways during PTI signaling

**Fig. 5.**
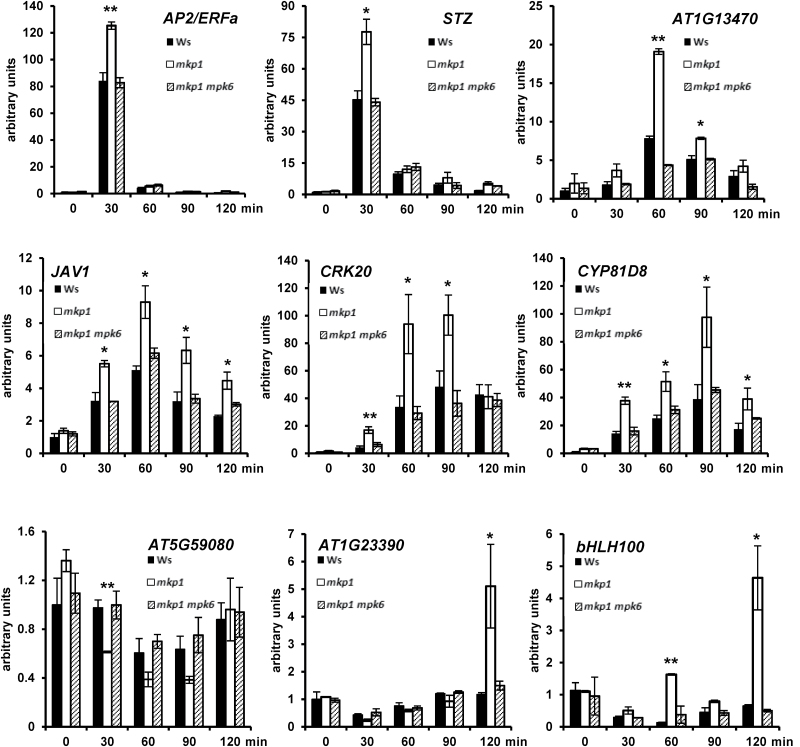
Independent qRT-PCR verification of transcripts that are both MKP1 dependent and MPK6 dependent. Transcript levels of candidate genes measured by quantitative RT-PCR from 12-day-old seedlings treated with or without 1 μM elf26 for indicated time points. Transcript levels were normalized to *At2g28390* in each sample, then to transcript level at time 0 in Ws. Values are means±SE, *n*=3. Asterisks indicate significant differences between Ws and *mkp1* (*P<0.05, **P<0.01). Ws and *mkp1 mpk6* are not significantly different in any result. Data are pooled from three biological replicates.

**Fig. 6.**
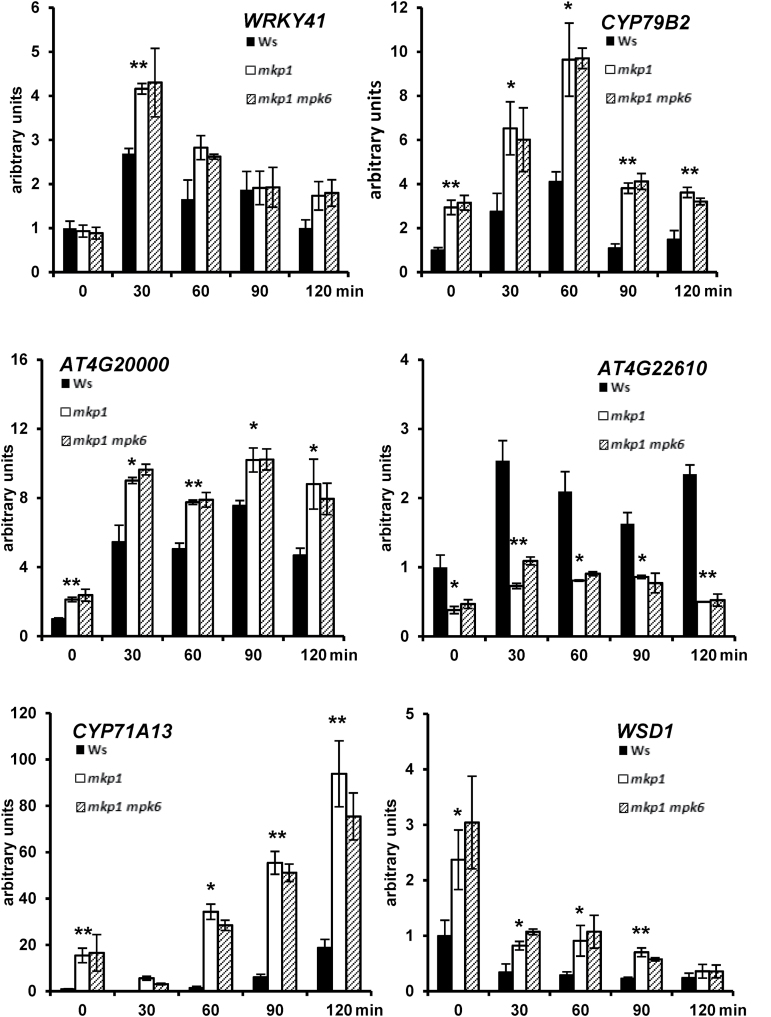
Independent qRT-PCR verification of transcripts that are MKP1 dependent but MPK6 independent. Transcript levels of candidate genes measured by quantitative RT-PCR from 12-day-old seedlings treated with or without 1 μM elf26 for indicated time points. Transcript levels were normalized to *At2g28390* in each sample, then to transcript level at time 0 in Ws. Values are means±SE, *n*=3. Asterisks indicate significant differences between Ws and *mkp1* (*P<0.05, **P<0.01). Results from *mkp1* and *mkp1 mpk6* are not significantly different in any experiment. Data are pooled from three biological replicates.

**Fig. 7.**
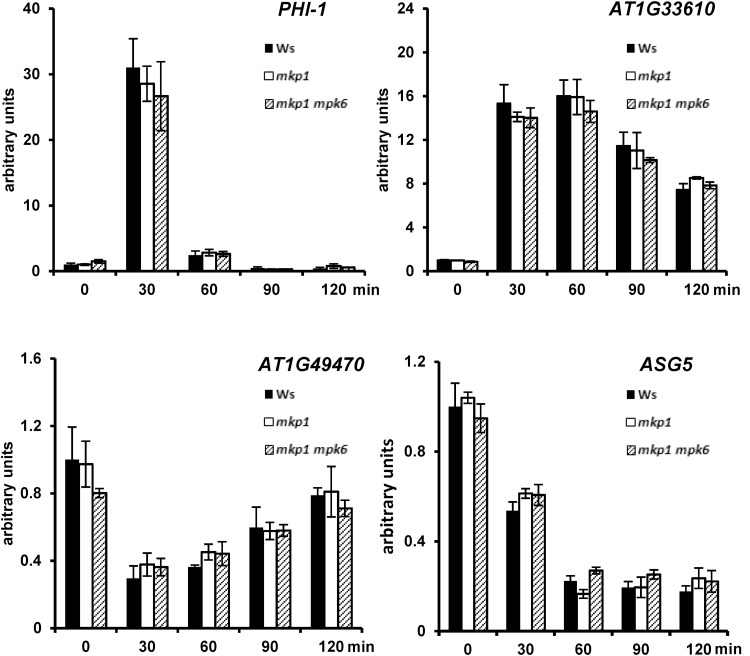
Independent qRT-PCR verification of transcripts that are MKP1 independent. Transcript levels of candidate genes measured by quantitative RT-PCR from 12-day-old seedlings treated with or without 1 μM elf26 for indicated time points. Transcript levels were normalized to *At2g28390* in each sample, then to transcript level at time 0 in Ws. No significant difference was detected between any genotype. Values are means±SE, *n*=3. Data are pooled from three biological replicates.

By comparing the transcriptional changes in three different genotypes (Ws, *mkp1*, and *mkp1 mpk6* mutants), we were able to genetically separate the elf26-responsive transcripts into MKP1-independent, MKP1-dependent/MPK6-dependent, and MKP1-dependent/MPK6-independent categories. Temporal profile clustering analysis further separated the transcripts into different clusters with similar transcriptional accumulation patterns. Based on both genetic separation and expression kinetics, we have generated a novel set of candidates that can serve as marker genes representative of different regulatory pathways during PTI signaling. For validation, we selected nine MKP1-dependent/MPK6-dependent (Fig. 5), six MKP1-dependent/MPK6-independent (Fig. 6), and four MKP1-independent (Fig. 7) candidates representative of different termporal accumulation patterns. qRT-PCR was used to measure the expression of 19 genes in response to elf26 in the same genotypes treated for RNAseq. We also extended the time course from 0.5 to 2 h following elf26 treatment to potentially clarify patterns of transcript accumulation that might have been missed in the RNAseq analysis at only 30 and 90 min post-elicitation.

Overall, the results from qRT-PCR analyses were consistent with that from RNAseq, thereby validating the large-scale analysis. Two conclusions arise from the results of qRT-PCR that are consistent with our conclusions from the RNAseq dataset. First, there are diverse accumulation patterns for elf26-responsive transcripts within all different genotype-specific response categories (Figs 5–7). Second, transcripts with similar accumulation patterns have different genetic requirements. For instance, both *AP2*/*ERFa* and *PHI-1* (*PHOSPHATE-INDUCED 1*) were rapidly and strongly induced at 30 min but rapidly returned to basal levels by 90 min. However, *AP2*/*ERFa* belongs to the MKP1-dependent/MPK6-dependent category (Fig. 5), whereas *PHI-1* was MKP1 independent (Fig. 7).

In addition to validating our RNAseq results, the extended qRT-PCR time course provided additional information that helped to refine gene expression patterns. For example, *AT1G13470* and *JAV1* (*JASMONATE-ASSOCIATED VQ MOTIF GENE 1*), marker genes for the MKP1-dependent/MPK6-dependent pathway, were initially identified from the RNAseq data as being rapidly induced at 30 min with the induction either sustained or amplified at 90 min. However, when assayed over an extended time course by qRT-PCR, we observed that both *AT1G13470* and *JAV1* showed a peak expression at 60 min that actually declined by 90 min (Fig. 5). Similarly, *CYP79B2* in the MKP1-dependent/MPK6-independent pathway appeared to have a peak expression at 30 min from RNAseq analysis, but actually peak expression was observed at 60 min by qRT-PCR (Fig. 6). We also observed two marker genes (*AT1G23390* and *bHLH100*) within the MKP1-dependent/MPK6-dependent category that strongly accumulated in *mkp1* at 2 h post-elicitation, whereas no accumulation compared with the 0 min was observed in wild type or *mkp1 mpk6* double mutant (Fig. 5). This last result indicates that over longer times, the *mkp1* mutation results in the expression of genes that are not normally induced by elf26.

A next step will be to identify the transcription factors responsible for regulating these transcripts during PAMP responses. Initial candidates for some of the MKP1-dependent transcripts include WRKY18, WRKY33, and WRKY40, which were shown by ChIP analyses (Birkenbihl *et al.*, 2017) to interact with promoters from 15–32% of the genes (data and individual genes are summarized in Supplementary Table S9). Future genetic experiments introducing mutations lacking these WRKY factors into the *mkp1* mutant will be necessary to begin to dissect these early signaling pathways.

### Separation of previously identified PTI signaling components downstream MPK6

MAPK cascades are important components for integrating responses to extracellular stimuli, and a number of signaling components or modules downstream of MAPK cascades have been described (Li *et al.*, 2016). WRKY22 and WRKY29 are two transcription factors induced by flg22 treatment that are downstream of a flg22-activated MEKK1/MTK–MKK4/MKK5–MPK3/MPK6 cascade (Asai *et al.*, 2002). WRKY33 is a direct substrate for MPK3 and MPK6, and plays an essential role in immunity against the necrotrophic fungus *Botrytis* (Mao *et al.*, 2011). PAMP-activated MPK6 also phosphorylates BES1, a transcription factor involved in brassinosteroid (BR) signaling pathway, which positively regulates the resistance to bacteria and PTI gene expression (Kang *et al.*, 2015).

Multiple signaling components involved in ethylene signaling are also downstream of MPK3 and MPK6. Transcripts encoding ACS2 and ACS6, two isoforms of the rate-limiting enzyme in ethylene biosynthesis, are induced upon pathogen attack in an MPK3- and MPK6-dependent manner through phosphorylation of WRKY33 (Li *et al.*, 2012). The ethylene response factor ERF104, a regulator of basal immunity, is also a substrate of PAMP-activated MPK6 (Bethke *et al.*, 2009). Similarly, ERF6 is phosphorylated by MPK3 and MPK6 upon *Botrytis* infection (Meng *et al.*, 2013). Both the protein stability and transcript encoded by *ERF6* are regulated by MPK3/MPK6 activation (Meng *et al.*, 2013).

Although previous studies placed all these signaling components downstream of MPK6, our transcriptome analysis indicates that they can be separated by their requirement for MKP1, indicating that they are within different sub-pathways of MAP kinase signaling. For instance, the transcripts of *WRKY22*, *WRKY29*, and *BES1* are all MKP1 independent at either 30 or 90 min post-elicitation (Table 2). However, *WRKY33* switches from the MKP1-independent category at 30 min to MKP1-dependent category at 90 min (Table 2). For the components involving in ethylene signaling, *ACS2* transcript accumulation remains independent of MKP1 at both 30 and 90 min, whereas *ACS6* shifts from MKP1 independent at 30 min to MKP dependent at 90 min (Table 2). *ERF104* and *ERF6* are also distinct, with *ERF104* being MKP1 dependent while *ERF6* is MKP1 independent (Table 2). Therefore, our transcriptome analysis provides important additional information that further distinguishes components that had previously been clustered together as merely downstream of MPK6 into those that are or are not MKP1 dependent.

**Table 2.** Comparison of previously identified MPK6 substrates and/or downstream signaling components based on requirement of MKP1-regulation in response to elf26

Gene	Gene identifier	30 min	90 min
*WRKY22*	AT4G01250	MKP1 independent	MKP1 independent
*WRKY29*	AT4G23550	MKP1 independent	MKP1 independent
*WRKY33*	AT2G38470	MKP1 independent	MKP1 dependent MPK6 independent
*BES1*	AT4G18890	MKP1 independent	MKP1 independent
*ACS2*	AT1G01480	MKP1 independent	MKP1 independent
*ACS6*	AT4G11280	MKP1 independent	MKP1 dependent partially MPK6 dependent
*ERF104*	AT5G61600	MKP1 dependent MPK6 dependent	Not PAMP responsive
*ERF6*	AT4G17490	MKP1 independent	Not PAMP responsive

### Identification of new potential signaling components downstream of MPK6

Our transcriptome analysis has identified a novel set of transcripts that require the regulation of MKP1, with or without the contribution of MPK6. This set of transcripts can contribute to understanding PAMP-induced signaling by delineating the positions of putative downstream components of MPK6 in the context of MKP1-dependent signaling. That is, if MPK6 is required for the correct transcript accumulation pattern, MPK6 substrates or downstream signaling components must also be required to connect MPK6 to the transcript changes. The use of molecular phenotypes (e.g. transcript accumulation) is likely to be more efficient than using biological phenotypes such as resistance or growth inhibition. In this regard, if the output(s) of MPK6 kinase activity is integrated through multiple signaling pathways, analysis of knockout mutants for individual substrates may not result in easily detectable biological phenotypes as these outputs tend to be more variable. However, screens based on molecular phenotypes are likely to be more sensitive to quantitative contributions of individual substrates or downstream signaling components. Therefore, the MPK6-dependent gene markers identified from our studies are likely to be more refined readouts for separating substrates/signaling components downstream of MPK6 within the MKP1-dependent pathway. By the same logic, the improved list of potential pathway markers for MKP1-independent and MKP1-dependent/MPK6-independent pathways will provide more refined tools for phenotyping mutants in diverse PAMP-induced signaling pathways.

## Supplementary data

Supplementary data are available at *JXB* online

Fig. S1. Genotyping of *mkp1* and *mkp1 mpk6* mutants.

Table S1. qRT-PCR primers used in this study.

Table S2. RNAseq reads and mapping statistics.

Table S3. Numbers of PAMP-responsive transcripts at 30 and 90 min post-elf26 elicitation.

Table S4. Comprehensive list of elf26-responsive genes in Ws and *mkp1* at 30 and 90 min post-elicitation including normalized expression values, log2-fold change (Fc), *q* values and annotations.

Table S5. Comprehensive list of MKP1-dependent and MKP1-independent genes at 30 and 90 min post-elicitation including normalized expression values, log2-fold change (Fc), *q* values and annotations.

Table S6. Analysis of Gene Ontology (GO) enrichment in MKP1-dependent genes and elf26-regulated genes.

Table S7. Comprehensive list of MKP1-dependent, MPK6-dependent, and MKP1-dependent MPK6-independent genes at 30 and 90 min post-elicitation including normalized expression values, log2-fold change (Fc), *q* values and annotations.

Table S8. Comprehensive list of genes in each clusters including the normalized expression values at different time points in different genotypes and annotations.

## Supplementary Material

Supplementary figure S1 tables S1-S3

Supplementary table S4

Supplementary table S5

Supplementary table S6

Supplementary table S7

Supplementary table S8

Supplementary table S9
