# Supplementary material for: Genetic dissection of Arabidopsis MAP kinase phosphatase 1-dependent PAMP-induced transcriptional responses
Source: J Exp Bot. 2017 Oct 13;68(18):5207–20. doi: 10.1093/jxb/erx335 (PMC5853853; doi:10.1093/jxb/erx335)
Supplement: Supplementary figure S1 tables S1-S3 [file erx335_suppl_supplementary_figure_s1_tables_s1-s3.pdf]

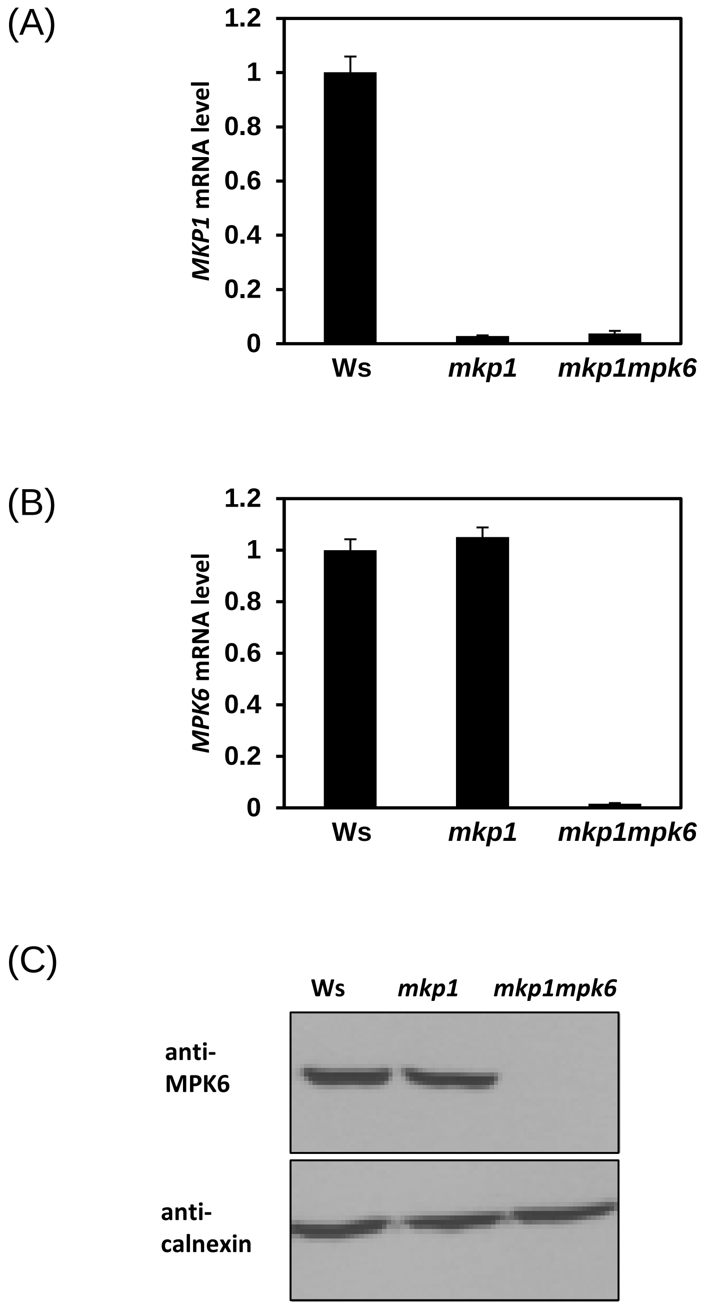


**Figure S1. Genotyping of *mkp1* and *mkp1mpk6* mutants**

(A) qPCR analysis of *MKP1* transcripts in total RNA from untreated 12-day-old seedlings. Each sample was RNA pooled from 6-8 seedlings and three samples per genotype were analyzed. Graphed are means ±SE, n=3.

(B) qPCR analysis of *MPK6* transcripts in total RNA from untreated 12-day-old seedlings. Each sample was RNA pooled from 6-8 seedlings and three samples per genotype were analyzed. Graphed are means ±SE, n=3.

(C) Immunoblot analysis of protein extracts from untreated 12-day-old seedlings using specific anti-MPK6 (upper panel) or anti-calnexin (lower panel) antibodies for loading control.

**Supplementary Table S1. qRT-PCR primers used in this study**

| Gene | AGI number | Primer |
|---|---|---|
| *MKP1* | At3g55270 | Fw: 5'-CCTGAAAGGATCTCTGAAGTTGCC-3'<br>Rev: 5'-CCGTTTCTAATACCCTCCAGATTG-3' |
| *MPK6* | At2g43790 | Fw: 5'-CTCGTTGCACGACATAAGCG-3'<br>Fw: 5'-CGCCTCGCGGTAGATTAGTT-3' |
| *AP2/ERFa* | At5g51190 | Fw: 5'-TTAACGCCATCAAGTTGGAAG-3'<br>Rev: 5'-TCCAAGAGATGGACAAGGAGA-3' |
| *STZ* | At1g27730 | Fw: 5'-GCCACGTTAGCAGTAGCCA-3'<br>Rev: 5'-ACCGGAAAGTCAAACCGAGG-3' |
| *At1g13470* | At1g13470 | Fw: 5'-ACAAGCGTTGTGGTAGGAAA-3'<br>Rev: 5'-AACCTCCTCCCATCTTTGTAGA-3' |
| *JAV1* | At3g22160 | Fw: 5'-GGCTTTCGGGTCCGGTAATA-3'<br>Rev: 5'-CGCGTGAGGCTGGAAATTAT-3' |
| *CRK20* | At4g23280 | Fw: 5'-TTCAACGTTGGAAAAGGCGGA-3'<br>Rev: 5'-TTTTCTCTTAGCGCGGCGG-3' |
| *CYP81D8* | At4g37370 | Fw: 5'-GGACTGGCTCATCGGCTTAT-3'<br>Rev: 5'-GGCATTGTAACACCTTTGCC-3' |
| *At5g59080* | At5g59080 | Fw: 5'-CATGGCTCACAAGCTCAACG-3'<br>Rev: 5'-TTGGTTGGTGGTGGATCGAG-3' |
| *At1g23390* | At1g23390 | Fw: 5'-TTATGGCTGGGATCATCGGA-3'<br>Rev: 5'-TCTCCAAATACGTCTCAGGCA-3' |
| *bHLH100* | At2g41240 | Fw: 5'-CAGAGCTGCAAGAGCAAGTT-3'<br>Rev: 5'-ACGCATAGCTTGTAACCCCTT-3' |
| *WRKY41* | At4g11070 | Fw: 5'-CACACAACACAATCCAACGATAG-3'<br>Rev: 5'-ACTCTTCGCTTCTCGGATTTC-3' |
| *CYP79B2* | At4g39950 | Fw: 5'-CAGGATGGCCGATCATTGGA-3'<br>Rev: 5'-GCTGCTTCATGATGCTGTGG-3' |
| *At4g20000* | At4g20000 | Fw: 5'-TGGATTTCGGGTCTCCCACT-3'<br>Rev: 5'-GCTCGTTACTCACGTGTTGG-3' |
| *At4g22610* | At4g22610 | Fw: 5'-TGTCTGCATCTGCGATTCCA-3'<br>Rev: 5'-GATAGTCCGCTCCGCAAAGG-3' |
| *CYP71A13* | At2g30770 | Fw: 5'-CGATTCCTCAAACGAACCGC-3'<br>Rev: 5'-TACCTGAGACTGAGGGACCG-3' |
| *WSD1* | At5g37300 | Fw: 5'-TGGCGAAGGGTTCAAAGTGT-3'<br>Rev: 5'-TCCATCGTAGCTTTGGCTCG-3' |
| *PHI-1* | At1g35140 | Fw: 5'-GACTCAGTCTCTCCCTCGGA-3'<br>Rev: 5'-TAGCTTTGGCCACCTTTTGC-3' |
| *At1g33610* | At1g33610 | Fw: 5'-CACGGTGGCTGGTCTACAAA-3'<br>Rev: 5'-GGAGAAAGTGGAGAGCCACA-3' |
| *At1g49470* | At1g49470 | Fw: 5'-ATCCTTCTCTGCGGCTCTATT-3'<br>Rev: 5'-ACCCCATGTTCAAGAACCAGC-3' |
| *ASG5* | At1g20650 | Fw: 5'-ATCGACCGTTCATAGGTGACAT-3'<br>Rev: 5'-TTCTTGAAATCTCTGGTGACGGT-3' |

**Supplementary Table S2. RNAseq reads and mapping statistics**

| Sample | Total reads | Reads mapped | Percentage mapped |
|---|---|---|---|
| Ws_0_r1 | 28464813 | 25455616 | 89.43% |
| Ws_0_r2 | 28084244 | 25135755 | 89.50% |
| Ws_0_r3 | 25472221 | 22758060 | 89.34% |
| Ws_30_r1 | 27114807 | 24271396 | 89.51% |
| Ws_30_r2 | 23385907 | 20962479 | 89.64% |
| Ws_30_r3 | 28232682 | 25264976 | 89.49% |
| Ws_90_r1 | 25033996 | 22410590 | 89.52% |
| Ws_90_r2 | 35682508 | 31937051 | 89.50% |
| Ws_90_r3 | 27755215 | 24804288 | 89.37% |
| *mkp1*_0_r1 | 23715130 | 21133020 | 89.11% |
| *mkp1*_0_r2 | 26512288 | 23646642 | 89.19% |
| *mkp1*_0_r3 | 22533079 | 20078407 | 89.11% |
| *mkp1*_30_r1 | 27287352 | 24358068 | 89.27% |
| *mkp1*_30_r2 | 23754657 | 21200605 | 89.25% |
| *mkp1*_30_r3 | 25880226 | 23044514 | 89.04% |
| *mkp1*_90_r1 | 26511655 | 23683342 | 89.33% |
| *mkp1*_90_r2 | 39427945 | 35196352 | 89.27% |
| *mkp1*_90_r3 | 21840801 | 19495129 | 89.26% |
| mm6_0_r1 | 26083896 | 23256381 | 89.16% |
| mm6_0_r2 | 25944302 | 23159288 | 89.27% |
| mm6_0_r3 | 25541526 | 22809792 | 89.30% |
| mm6_30_r1 | 27486329 | 24520879 | 89.21% |
| mm6_30_r2 | 31620116 | 28212006 | 89.22% |
| mm6_30_r3 | 26158912 | 23317215 | 89.14% |
| mm6_90_r1 | 24185982 | 21561388 | 89.15% |
| mm6_90_r2 | 37488452 | 33420508 | 89.15% |
| mm6_90_r3 | 26208281 | 23356334 | 89.12% |
| total | 737407322 | 658450081 | 89.29% |

mm6: *mkp1 mpk6*

**Supplementary Table S3. Number of PAMP responsive transcripts at 30 min and 90 min post elf26 elicitation**

| | 30min | 90min |
|---|---|---|
| elf26 up-regulated transcripts | 2114 | 2995 |
| elf26 down-regulated transcripts | 781 | 2994 |
| Total | 2895 | 5989 |
